# Nootkatone Alleviates Type 2 Diabetes in db/db Mice Through AMPK Activation and ERK Inhibition: An Integrated In Vitro and In Vivo Study

**DOI:** 10.3390/molecules30102111

**Published:** 2025-05-09

**Authors:** Yingjie Li, Linlin Zheng, Mimi Chen, Ruodi Li, Yansu Yu, Lu Qiao, Jialu Liu, Xiaopo Zhang, Yong Zhang, Yuxin Zhang, Wei Zheng

**Affiliations:** 1 School of Pharmacy, Harbin University of Commerce, Harbin 150028, China; maniaclyj@icloud.com; 2 Department of Pharmacology, College of Basic Medicine and Life Sciences, Hainan Medical College, Haikou 571199, China; 3 Hainan Academy of Medical Sciences, Haikou 571199, China; 4 School of Pharmacy, Hainan Medical College, Haikou 571199, China

**Keywords:** nootkatone, diabetes, liver, pancreatic, network pharmacology, db/db mice

## Abstract

Type 2 diabetes mellitus (T2DM) is a common chronic metabolic disorder that imposes a substantial healthcare burden globally. Recent advances highlight the potential of natural products in ameliorating T2DM. In this study, we investigated the therapeutic efficacy of nootkatone (Nok), a natural sesquiterpene ketone, in T2DM and elucidated its underlying mechanisms. In vivo experiments demonstrated that Nok administration markedly improved dysregulated glucose metabolism and ameliorated serum biochemical abnormalities in db/db mice. Leveraging a network pharmacology-based approach, we identified putative molecular targets of Nok. Subsequent in vitro analyses revealed that Nok significantly enhanced glucose consumption in cultured cells. Mechanistically, Nok robustly activated AMP-activated protein kinase (AMPK) while suppressing mitogen-activated protein kinase (MAPK) signaling. Western blot validation further indicated that Nok downregulated the phosphorylation of MAPK1/3 (ERK2/1), attenuating MAPK pathway activation and thereby alleviating metabolic dysfunction-associated fatty liver disease (MAFLD) progression in the diabetic model. Collectively, our findings suggest that Nok exerts anti-diabetic effects via dual modulation of AMPK activation and MAPK inhibition, effectively restoring metabolic homeostasis and mitigating inflammation in T2DM. This study positions Nok as a promising natural compound for therapeutic intervention in T2DM and associated metabolic disorders.

## 1. Introduction

Type 2 diabetes mellitus (T2DM) is a chronic metabolic disorder characterized by hyperglycemia, relative insulin deficiency, and insulin resistance [1]. This condition often leads to multiple complications such as diabetic retinopathy, nephropathy, cardiovascular diseases, and neuropathy, which are significant causes of morbidity and mortality in diabetic patients [2]. Current pharmacological treatments for T2DM include sulfonylureas, biguanides, α-glucosidase inhibitors, thiazolidinediones, and insulin. While these medications effectively control blood glucose levels, they can also cause side effects such as hepatotoxicity, hypoglycemia, and edema, which may limit their tolerability for some patients [3]. Therefore, there is an urgent need for the development of new antidiabetic drugs with better efficacy and safety profiles.

*Alpinia oxyphylla*, a traditional medicinal herb native to Hainan Island, has been historically used to treat conditions such as enuresis, frequent urination, premature ejaculation, abdominal pain from spleen cold, and diarrhea [4]. The herb is an integral part of Hainan’s traditional medicine. The primary bioactive compounds of *Alpinia oxyphylla* include sesquiterpenoids, diarylheptanoids, flavonoids, volatile oils, and steroids, which have shown various pharmacological activities such as antidiuretic, cognitive enhancement, antimicrobial, antitumor, neuroprotection, and antioxidative stress [5].

Nootkatone (Nok), a sesquiterpenoid ketone, is a key bioactive component of *Alpinia oxyphylla* [6], grapefruit, and other citrus fruits. It is widely used in the flavoring and fragrance industries due to its distinctive grapefruit, orange, and tropical fruit aroma. Research has demonstrated that Nok enhances the sensitivity of non-small cell lung cancer A549 cells to doxorubicin [7], protects against chronic kidney injury, and exhibits anxiolytic and antidepressant properties [8]. These pharmacological effects are related to its activation of AMPK, a highly conserved intracellular protein kinase that regulates energy homeostasis and is considered a crucial target for treating metabolic disorders such as diabetes, dyslipidemia, fatty liver, and obesity [9,10,11]. Our previous research showed that Nok can significantly improve glucose and lipid metabolism disorders and enhance glucose tolerance in MAFLD animal model. Therefore, we hypothesize that Nok may also have beneficial therapeutic effects for T2DM.

## 2. Results

### 2.1. Nok Improves Glucose and Lipid Metabolism and Reduces Body Weight in db/db Mice

**Figure 1:**
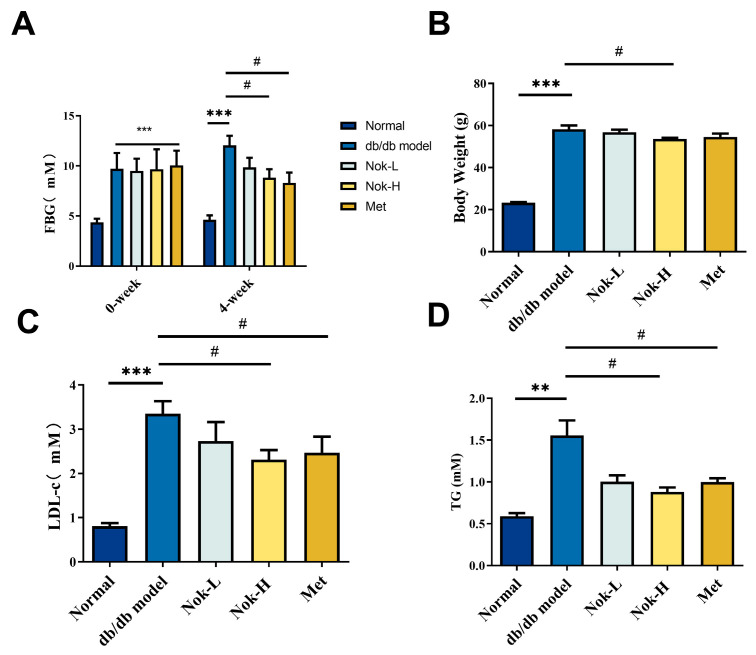
The effects of Nok on fasting blood glucose, blood lipids, and body weight in mice. (**A**) Fasting blood glucose (FBG); (**B**) body weight (**C**); low-density lipoprotein (LDL); (**D**) triglycerides (TG). All data are presented as mean ± S.E.M., with n = 6. ** *p* < 0.01 or *** *p* < 0.001 vs. Normal, # *p* < 0.05 vs. db/db model.

To investigate the efficacy of Nok on T2DM, we first examined the effects of Nok on glucose and lipid metabolism as well as changes in body weight. As illustrated in Figure 1, fasting blood glucose (FBG) levels in db/db diabetic mice were significantly higher than those in C57BL/6J mice (Figure 1A). Following four weeks of Nok administration, FBG levels in the Nok-treated db/db mice decreased in a dose-dependent manner. Notably, the 100 mg/kg Nok-H group exhibited a significant reduction in FBG levels compared to the untreated db/db model group, achieving a 26.74% decrease in blood glucose concentration. In contrast, FBG levels in the untreated db/db model group increased significantly from baseline, whereas all Nok-treated groups displayed reductions (Figure 1A). The glucose-lowering efficacy of Nok was comparable to that observed in the 200 mg/kg metformin-treated group (Figure 1A).

In terms of body weight, the Nok treatment groups showed varying degrees of weight reduction compared to the db/db model group, with the 100 mg/kg Nok-H group showing a significant weight reduction effect (Figure 1B). Lipid profile analysis indicated that serum LDL-c and TG levels were significantly higher in the db/db model group compared to the normal C57BL/6J mice (Figure 1C,D). However, after treatment with 100 mg/kg Nok, serum LDL-c and TG levels decreased significantly (Figure 1C,D), with lipid-lowering effects comparable to those of metformin (Figure 1C,D).

### 2.2. Nok Improves Glucose Tolerance and Insulin Resistance in db/db Diabetic Mice

**Figure 2:**
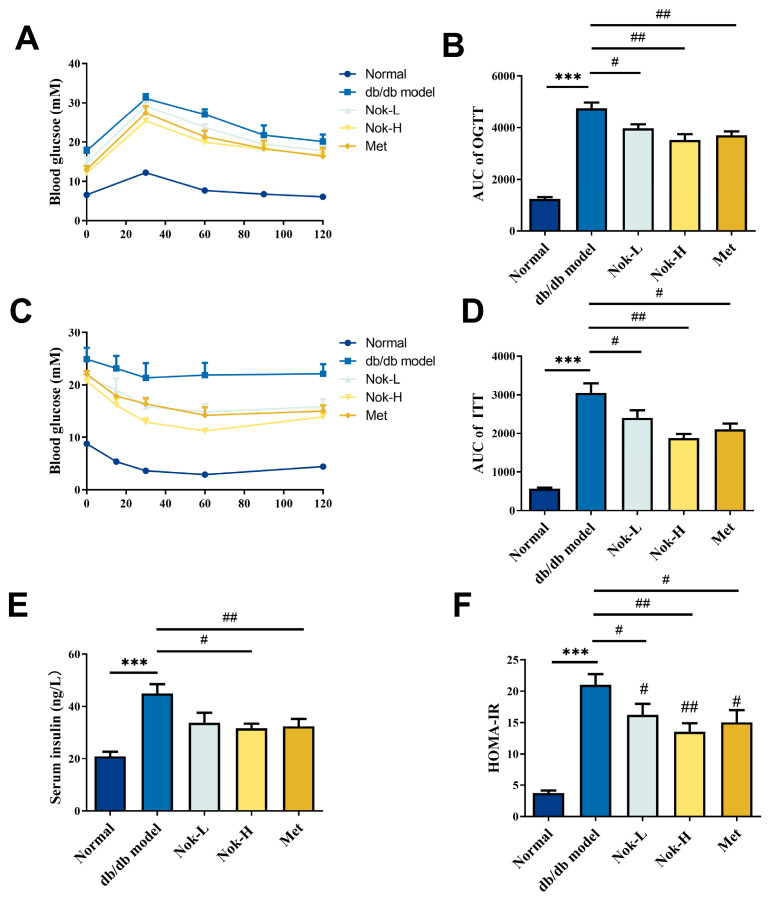
Effect of Nok on glucose tolerance and insulin resistance in db/db diabetic mice. (**A**) Oral glucose tolerance test (OGTT); (**B**) area under the curve (AUC) for OGTT; (**C**) insulin tolerance test (ITT); (**D**) AUC for ITT; (**E**) serum insulin concentration; (**F**) homeostasis model assessment of insulin resistance (HOMA-IR). All results are expressed as mean ± S.E.M., n = 6. *** *p* < 0.001 vs. Normal, # *p* < 0.05 or ## *p* < 0.01 vs. db/db model.

Glucose tolerance and insulin resistance are key indicators and contributors to the development of T2DM. To further assess the effects of Nok on these parameters in db/db diabetic mice, we conducted oral glucose tolerance tests (OGTT) and insulin tolerance tests (ITT), alongside measurements of serum insulin levels and calculation of the Homeostatic Model Assessment of Insulin Resistance (HOMA-IR). As shown in Figure 2A,B, blood glucose levels peaked 30 min after oral glucose administration in all mice and then gradually decreased. Compared to the C57BL/6J control group, the area under the curve (AUC) for the OGTT in the untreated db/db model group was significantly elevated, approximately 3.79-fold higher, reflecting impaired glucose tolerance. After four weeks of Nok treatment, glucose tolerance in the Nok-treated groups improved significantly compared to the untreated db/db model group, with AUC values decreasing in a dose-dependent manner (Figure 2B).

For ITT results (Figure 2C,D), blood glucose levels in the normal C57BL/6J mice significantly decreased after intraperitoneal insulin injection, reaching their lowest level around 60 min. In contrast, the untreated db/db model diabetic mice showed no significant decrease in blood glucose levels post-insulin injection, with AUC values significantly higher than those of the C57BL/6J group, approximately 5.6 times greater, indicating marked insulin resistance. However, in Nok-treated db/db mice, blood glucose levels significantly decreased after insulin injection, with a minimum value reached at 60 min, and AUC values significantly lower than those of the untreated db/db model mice, comparable to the Metformin-treated group (Figure 2C,D), suggesting that Nok markedly improves insulin resistance in db/db diabetic mice. Further analysis of serum insulin levels showed that insulin levels and HOMA-IR were significantly elevated in the db/db model group compared to the normal C57BL/6J group, indicating hyperinsulinemia and insulin resistance (Figure 2E,F). However, Nok treatment at a dose of 100 mg/kg significantly reduced insulin levels and HOMA-IR values, comparable to those in the Metformin-treated group (Figure 2E,F).

### 2.3. Nok Significantly Reduces Inflammation in db/db Diabetic Mice

**Figure 3:**
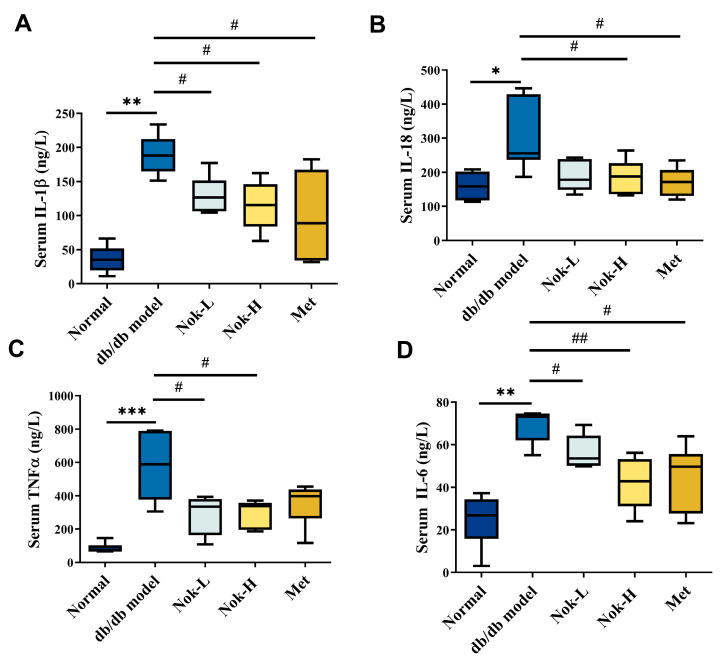
Effect of Nok on serum levels of inflammatory factors in mice. (**A**) IL-1β; (**B**) IL-18; (**C**) TNFα; (**D**) IL-6. All results are presented as mean ± S.E.M., n = 6. *** *p* < 0.001, ** *p* < 0.01 * *p* < 0.05 vs. Normal, # *p* < 0.05 or ## *p* < 0.01 vs. db/db model.

Inflammation plays a pivotal role in the progression and pathogenesis of diabetes. To investigate the effects of Nok on inflammation in db/db diabetic mice, we quantified serum levels of key inflammatory cytokines across all experimental groups. As shown in Figure 3, levels of interleukin-1β (IL-1β), interleukin-6 (IL-6), interleukin-18 (IL-18), and tumor necrosis factor-α (TNF-α) were significantly elevated in the db/db diabetic model group compared to the C57BL/6J control group (Figure 3). These findings indicate that db/db diabetic mice exhibit pronounced inflammation, likely driven by metabolic dysregulation of glucose and lipid homeostasis. In contrast, Nok treatment reduced the levels of these inflammatory cytokines relative to the untreated db/db model group, with the most notable reductions observed in the high-dose (100 mg/kg) Nok group. Specifically, serum concentrations of IL-1β, IL-6, and IL-18 were significantly decreased in this group (Figure 3A,B,D). These results demonstrate that Nok effectively attenuates inflammation in db/db diabetic mice. The positive control, metformin (Met), similarly exerted significant anti-inflammatory effects (Figure 3).

### 2.4. Nok Improves Liver Function in db/db Diabetic Mice

**Figure 4:**
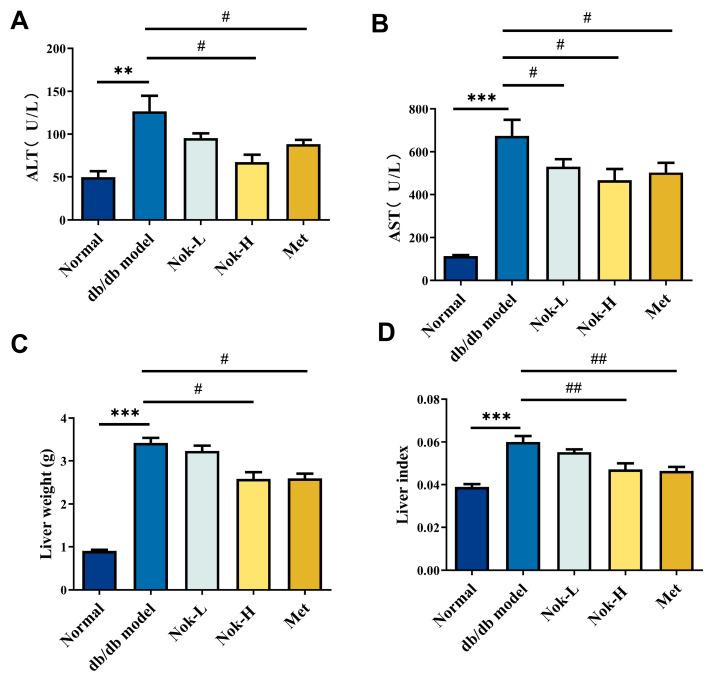
(**A**) Serum ALT levels; (**B**) serum AST levels; (**C**) liver weight; (**D**) liver index. All results are expressed as mean ± S.E.M., n = 6. ** *p* < 0.01 or *** *p* < 0.001 vs. Normal, # *p* < 0.05 or ## *p* < 0.01 vs. db/db model.

To evaluate liver function in mice, we measured serum levels of alanine aminotransferase (ALT) and aspartate aminotransferase (AST). Compared to the C57BL/6J control group, the db/db model group exhibited significantly elevated serum ALT and AST activities (Figure 4A,B). Additionally, liver mass and the liver index (liver mass/body weight ratio) were markedly higher in the db/db model group than in the C57BL/6J controls, indicative of liver enlargement and compromised hepatic function (Figure 4C,D). Following Nok treatment, serum ALT and AST activities were significantly reduced compared to the untreated db/db model group (Figure 4A,B). Similarly, treatment with 200 mg/kg metformin (Met), the positive control, significantly lowered ALT and AST activities, with effects comparable to those observed in the 100 mg/kg Nok-treated group (Figure 4A,B). Moreover, Nok treatment, particularly at the 100 mg/kg dose, significantly decreased liver mass and the liver index (Figure 4C,D). These findings suggest that Nok effectively mitigates diabetes-associated liver enlargement and enhances hepatic function.

### 2.5. Nok Improves Pathological Changes in the Liver and Pancreas of db/db Mice

**Figure 5:**
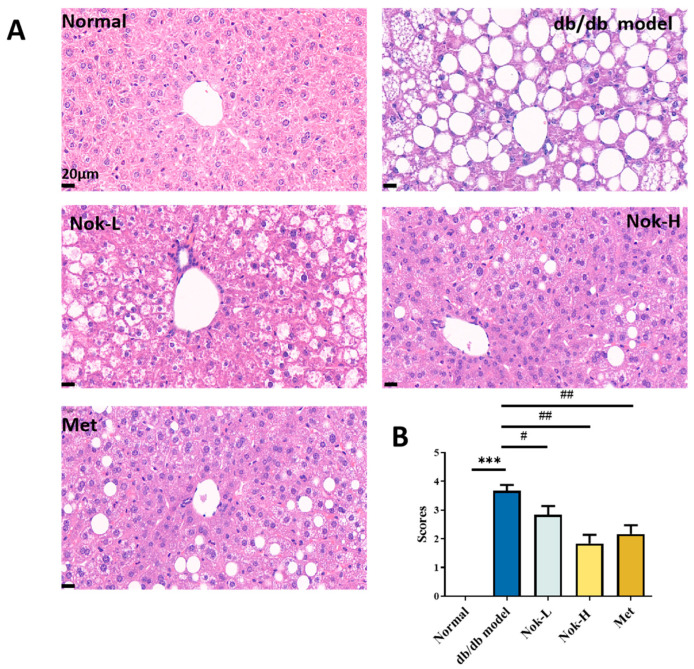
Effect of Nok on pathological changes in liver tissue of db/db diabetic mice. (**A**) HE staining of liver tissue; (**B**) pathological scoring results. Data are presented as mean ± SEM, n = 6. *** *p* < 0.001 vs. Normal, # *p* < 0.05 or ## *p* < 0.01 vs. db/db model.

Nok significantly reduces serum ALT and AST levels, decreases liver mass, and lowers the liver index, demonstrating pronounced hepatoprotective effects. To further elucidate these effects, we assessed pathological changes in liver tissue using hematoxylin and eosin (H and E) staining. As shown in Figure 5, liver sections from the db/db diabetic model group exhibited marked macrovesicular steatosis and cellular degeneration (Figure 5A). In contrast, the C57BL/6J control group displayed no evidence of steatosis or lipid accumulation, with the steatosis pathological score being significantly higher in the db/db model group than in the controls (Figure 5A). This suggests that liver enlargement in the db/db mice likely results from fat accumulation, corroborating the observed increases in liver mass and liver index. Nok treatment, however, significantly mitigated these pathological alterations, reducing both macrovesicular and microvesicular steatosis and substantially lowering pathological scores (Figure 5A,B). These findings indicate that Nok effectively protects liver function and ameliorates degeneration and lipid accumulation in hepatic tissue, providing further evidence of its hepatoprotective properties.

**Figure 6:**
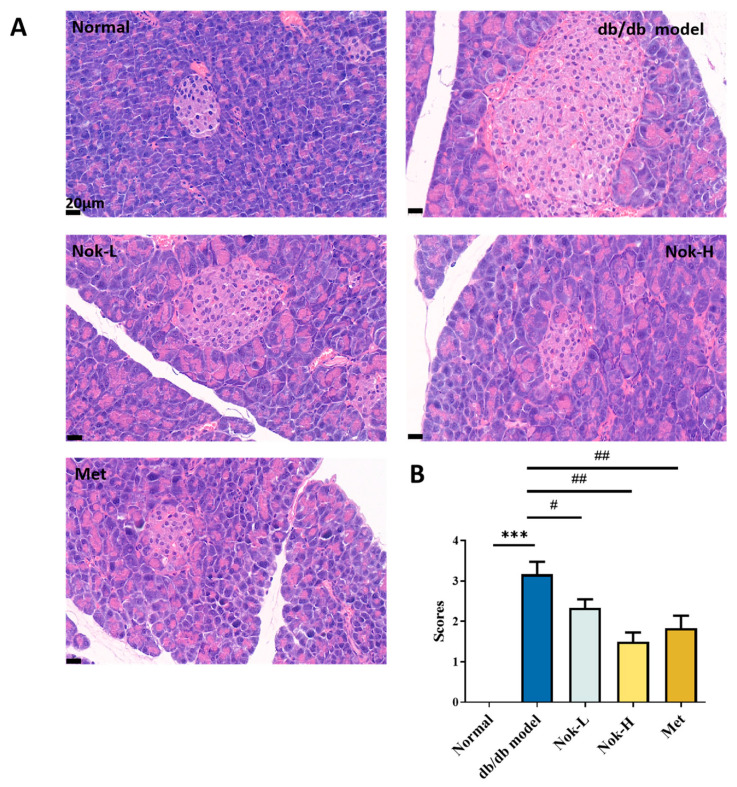
Effect of Nok on pathological changes in pancreatic tissue of db/db diabetic mice. (**A**) Histopathological results of HE staining; (**B**) pathological scoring results. Data are presented as mean ± SEM, n = 6. *** *p* < 0.001 vs. Normal, # *p* < 0.05 or ## *p* < 0.01 vs. db/db model.

We also examined pancreatic tissue using H and E staining. As shown in Figure 6, the pancreatic architecture of the C57BL/6J control group was well-defined, with islets appearing as spherical clusters of cells exhibiting regular morphology and a cord-like cellular arrangement (Figure 6). In contrast, the db/db diabetic model group displayed significantly enlarged islets, characterized by disorganized cellular structure, scattered vacuolar degeneration, and evidence of cell loss and collapse (Figure 6A). The pathological scores in the db/db group were significantly elevated compared to those in the C57BL/6J controls (Figure 6A). Following four weeks of Nok treatment, these pathological changes in the islets of db/db mice were markedly alleviated, with reductions in islet size, restoration of morphological integrity, and significant decreases in pathological scores (Figure 6A,B). The improvements in pancreatic histopathology observed in the 100 mg/kg Nok-treated group were comparable to those in the 200 mg/kg metformin (Met)-treated group.

### 2.6. The Effects of NOK on Glucose Metabolism and AMPK Activation

**Figure 7:**
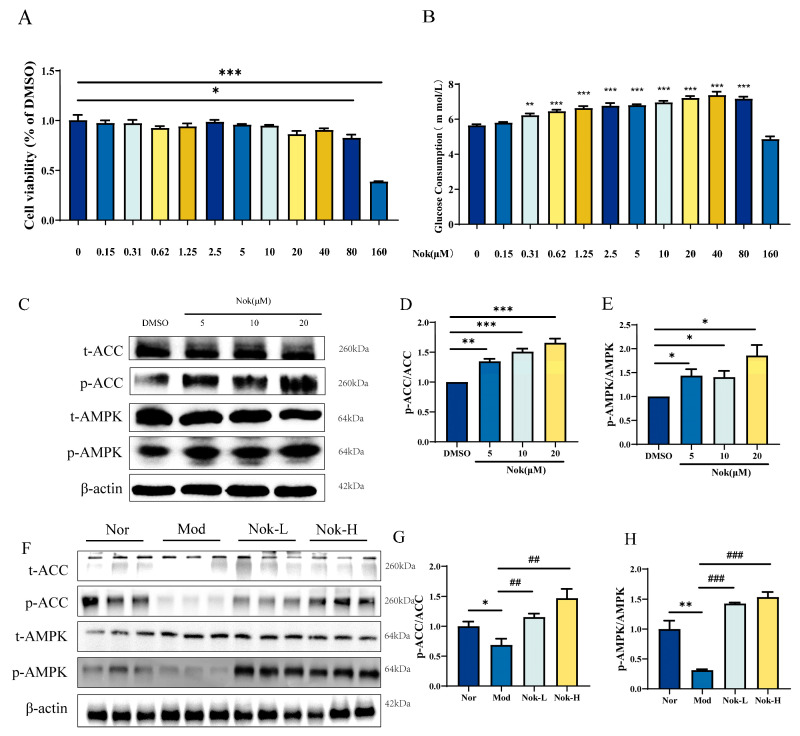
The Effects of NOK on glucose metabolism and AMPK activation. (**A**) Effects of Nok on cell viability in vitro; (**B**) glucose consumption; (**C**) Western blots were performed to analyze the levels of p-AMPK, AMPK, p-ACC and ACC in AML-12 cells. Examples of representative blots as above and fold changes of (**D**) p-ACC/t-ACC (**E**) p-AMPK/t-AMPK are shown by semiquantitative analyses; (**F**) Western blots were performed to analyze the levels of p-AMPK, AMPK, p-ACC and ACC in vivo. Examples of representative blots as above and fold changes of (**G**) p-ACC/t-ACC (**H**) p-AMPK/t-AMPK are shown by semiquantitative analyses. *** *p* < 0.001, ** *p* < 0.01 or * *p* < 0.05 vs. Normal or DMSO, ## *p* < 0.01 or ### *p* < 0.001 vs. db/db model.

To explore the effects of Nok on glucose metabolism and its underlying mechanism of action, we assessed its cytotoxicity and influence on cellular energy pathways in AML-12 hepatocytes. Nok exhibited no cytotoxicity in AML-12 cells at a concentration of 20 μM, whereas cell viability was significantly reduced at 80 μM (Figure 7A). Additionally, Nok increased glucose consumption in a dose-dependent manner (Figure 7B). Given the pivotal role of AMPK in regulating energy homeostasis and glucose metabolism, we investigated its activation in AML-12 cells. Western blot analysis demonstrated that Nok enhanced AMPK activation, as evidenced by elevated levels of phosphorylated AMPKα (Thr172) and its downstream target, phosphorylated acetyl-CoA carboxylase (ACC) (Ser79) (Figure 7C–G). These findings suggest that Nok may ameliorate type 2 diabetes mellitus (T2DM) by modulating glucose metabolism through the AMPK signaling pathway.

### 2.7. Network Pharmacology Prediction and Experimental Validation of Nok’s Targets

**Figure 8:**
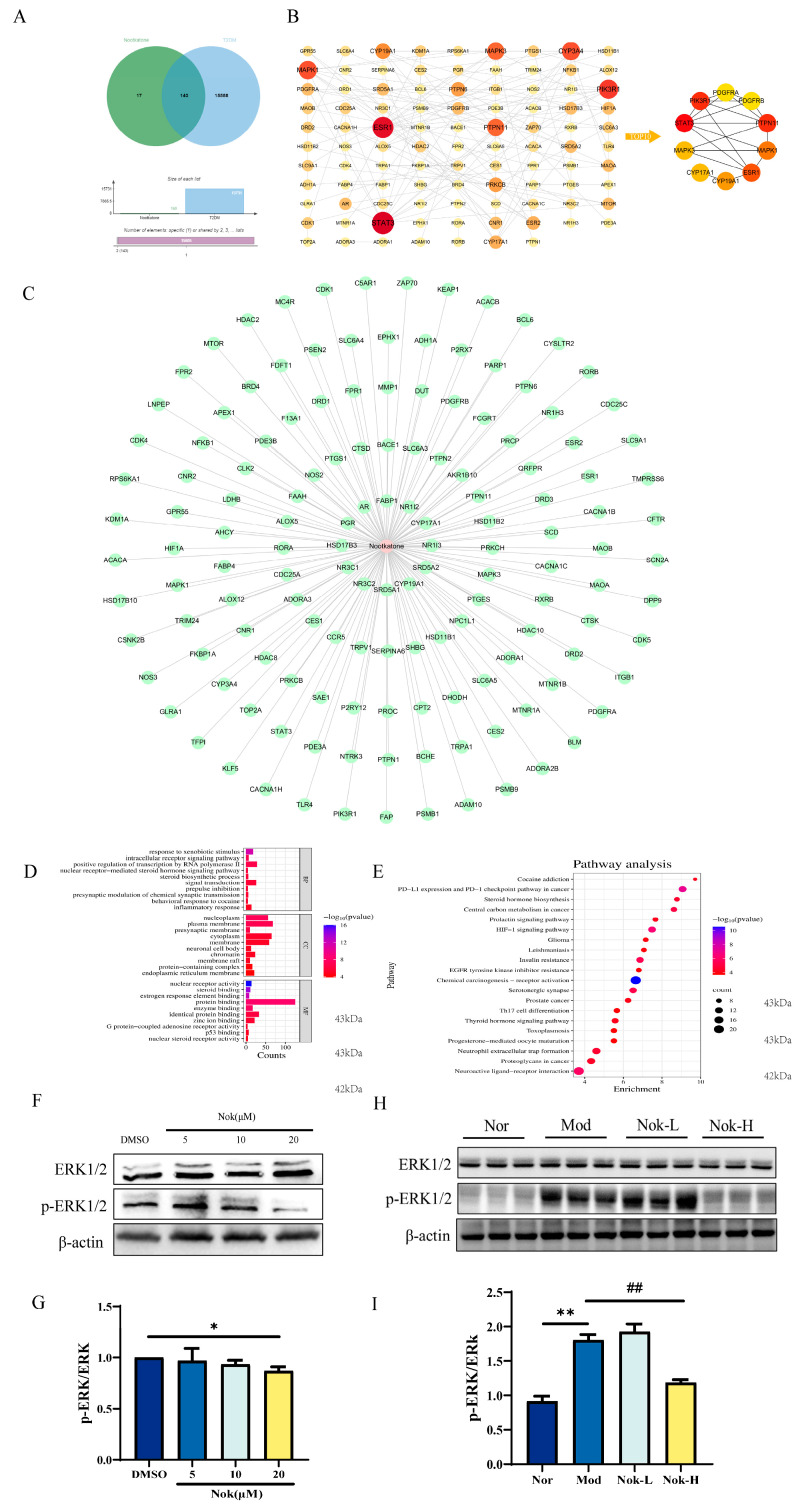
(**A**) Venn diagram illustrating the intersection between Nok-targeted proteins (green) and T2DM-associated proteins (blue). The 143 overlapping genes (cyan) represent potential therapeutic targets of Nok against T2DM; (**B**) cluster analysis of the protein-protein interaction (PPI) network. Node colors indicate functional modules identified via the MCODE algorithm; (**C**) network visualization of Nok-target protein interactions. Diamond nodes represent Nok-binding proteins, while circular nodes denote downstream effectors; (**D**) gene ontology (GO) enrichment analysis of overlapping genes, showing top five terms per category: biological process (BP), cellular component (CC), and molecular function (MF); (**E**) KEGG pathway analysis highlighting significantly enriched pathways (*p* < 0.05, FDR-corrected). Bar color intensity reflects −log10 (*p*-value); (**F**) representative Western blots of ERK and phosphorylated ERK (p-ERK) in AML-12 hepatocytes treated with Nok (50 μM, 24h); (**G**) densitometric quantification of p-ERK/ERK ratio from (**G**); Data normalized to β-actin (mean ± SEM, n = 6); (**H**) in vivo ERK phosphorylation analysis in liver tissues from db/db mice after four-week Nok treatment (50 or 150 mg/kg/day); (**I**) quantitative analysis of (**H**). ** *p* < 0.01 or * *p* < 0.05 vs. Normal; ## *p* < 0.01 vs. untreated db/db mice (two-way ANOVA with Tukey’s test).

To elucidate the therapeutic mechanisms of Nok in type 2 diabetes mellitus (T2DM), we employed an integrative network pharmacology approach. Comparative analysis of Nok-associated targets and T2DM-related proteins revealed 143 overlapping differentially expressed genes (DEGs), which were considered potential hub genes mediating Nok’s anti-diabetic effects. These DEGs were further analyzed using a protein–protein interaction (PPI) network, underscoring their functional significance in T2DM pathogenesis. To elucidate the mechanisms of Nok’s effects, we constructed a Nok target-T2DM network (Figure 8C). Gene function analysis of the 143 DEGs was performed using R software (version 3.5.1), with Gene Ontology (GO) annotation encompassing biological process (BP), cellular component (CC), and molecular function (MF) categories (Figure 8D). BP terms were predominantly enriched in inflammatory response, response to xenobiotic stimuli, signal transduction, and positive regulation of RNA polymerase II-mediated transcription. CC terms were associated with nucleoplasm, plasma membrane, and cytoplasm, while MF terms included protein binding, identical protein binding, and enzyme binding. Furthermore, Kyoto Encyclopedia of Genes and Genomes (KEGG) pathway enrichment analysis revealed significant associations between Nok and pathways such as insulin resistance and the hypoxia-inducible factor-1 (HIF-1) signaling pathway (Figure 8E). These results suggest that Nok’s therapeutic effects on T2DM are closely linked to these signaling pathways. Western blot analysis further demonstrated that Nok significantly suppressed the phosphorylation of extracellular signal-regulated kinase 1/2 (p-ERK1/2) both in vivo and in vitro (Figure 8F–I), indicating that its anti-inflammatory effects may be mediated, at least in part, through inhibition of the ERK signaling pathway.

## 3. Discussion

As the global prevalence of diabetes continues to rise, the burden on healthcare systems has become increasingly substantial. Although current anti-diabetic drugs offer some efficacy in glycemic control, long-term use can lead to side effects that render treatment intolerable for some patients. Consequently, the need for safer anti-diabetic therapies is pressing. In recent years, there has been growing interest in the potential of natural products and Traditional Chinese Medicine (TCM) in the development of anti-diabetic drugs. For example, resveratrol and laurolitsine have demonstrated significant efficacy in the treatment of T2DM [12]. The objective of this study was to systematically explore the potential therapeutic mechanisms of Nok in T2DM.

AMPK is a key energy sensor in cells, crucial for regulating glucose and lipid metabolism, which makes it a valuable target in T2DM treatment [13]. Activation of AMPK enhances insulin sensitivity by promoting glucose uptake in muscle and fat cells and inhibits lipogenesis by suppressing ACC [14,15], thereby reducing hepatic triglyceride accumulation and improving lipid profiles. Additionally, AMPK activation exerts anti-inflammatory effects by downregulating pro-inflammatory cytokines [16], which helps alleviate insulin resistance, a key factor in T2DM pathophysiology. Consequently, AMPK is an important therapeutic target for T2DM management [17], with AMPK activators such as metformin and berberine demonstrating notable effects on glycemic control and lipid metabolism. Building on our prior findings that Nok activates AMPK in MAFLD, this study confirmed that Nok’s effects on T2DM are likely mediated through AMPK activation, supporting its potential as a multi-functional therapeutic approach.

Inflammation plays a crucial role in the onset and progression of T2DM [18], with various signaling pathways contributing to the inflammatory environment that exacerbates insulin resistance and metabolic dysfunction. Among these, the role of the ERK signaling pathway is critical and cannot be overlooked. The ERK pathway not only mediates inflammatory responses but also influences insulin signaling, creating a complex link between inflammation and metabolic imbalance in T2DM [19]. Inhibiting the ERK signaling pathway has been shown to reduce inflammation and improve insulin sensitivity, making it a potential therapeutic target for T2DM. Drugs that modulate the ERK signaling pathway, such as resveratrol and PD98059, have demonstrated anti-inflammatory benefits in T2DM treatment [20]. These agents not only help control hyperglycemia but also alleviate the associated inflammation. In this study, we propose that Nok may exert its anti-T2DM effects through the modulation of the ERK signaling pathway, potentially by inhibiting inflammation that exacerbates insulin resistance. Our findings provide a foundation for further exploration of the molecular mechanisms by which Nok regulates the ERK pathway, potentially offering a novel strategy for developing therapeutic drugs for T2DM.

To explore potential molecular targets of Nok in T2DM, we used network pharmacology. Among the top 10 predicted targets, ERK1/2 emerged as a key target. Recent studies have identified the ERK signaling pathway as an important mechanism in the treatment of insulin resistance and T2DM. Inhibiting ERK has been shown to reduce obesity and prevent insulin resistance. Additionally, studies have demonstrated that diabetogenic factors can activate ERK, disrupting insulin signaling. Consequently, inhibiting ERK may represent a novel strategy for T2DM therapy. We confirmed Nok’s inhibitory effect on ERK1/2 both in vivo and in vitro, supporting the hypothesis that Nok exerts its effects on T2DM through ERK pathway inhibition. Nok exhibits distinct regulatory effects on ERK activity at low versus high doses in vivo, which may be explained by the biphasic dose-response hypothesis. Specifically, low-dose Nok treatment may transiently activate ERK through compensatory signaling mechanisms, while high-dose Nok achieves sustained pathway inhibition. While this study provides compelling evidence for Nok’s dose-dependent effects on ERK signaling and glycemic control, certain pharmacokinetic considerations warrant discussion. The apparent dichotomy between low-dose activation and high-dose inhibition of ERK may reflect not only pharmacodynamic mechanisms but also nonlinear pharmacokinetics. Although systematic PK(Pharmacikinetic) studies were beyond our current scope, existing literature suggests nootkatone undergoes extensive first-pass metabolism with potential autoinduction of CYP enzymes during chronic administration. This could lead to altered drug exposure over time, particularly at different dose levels. Future studies should incorporate comprehensive PK/PD(Pharmacodynamic) modeling to delineate whether the observed effects stem from true dose-response relationships or exposure-dependent pharmacokinetics. Such investigations would be invaluable for determining optimal dosing regimens in clinical translation. However, further research is needed to fully understand Nok’s molecular mechanisms, and these will be the focus of future investigations.

## 4. Materials and Methods

### 4.1. Reagents

Nootkatone (Nok) and metformin (Met) were purchased from Sigma (St. Louis, MO, USA, Cat No: W316620 and 317240). The ACCU-CHEK glucose meter and test strips were sourced from Roche Diagnostics (Basel, Switzerland) Co., Ltd. Enzyme-linked immunosorbent assay (ELISA) kits for mouse insulin were procured from the Institute of Biological Engineering. Alanine aminotransferase (ALT), aspartate aminotransferase (AST), low-density lipoprotein (LDL), and triglyceride (TG) assay kits were purchased from Beijing Jiuqiang Biological Technology Co., Ltd. (Beijing, China). D-(+)-glucose was obtained from Sigma (St. Louis, MO, USA). Recombinant human insulin injection was sourced from Beyotime Biotechnology (Shanghai, China). ERK1/2 and p-ERK1/2 (Thr202/Tyr204) Polyclonal antibody were purchased from proteintech (WuHan, China, Cat No: 51068-1-AP and 28733-1-AP). AMPK, p-AMPK, ACC and p-ACC antibody were purchased from Cell Signaling Technology (Danver, MA, USA, Cat NO: 2532S, 50081T, 3676T and 11818T). DMEM/F12, penicillin-streptomycin solution and fetal bovine serum were purchased from Gibco (California, USA, Cat NO: C11330500BT, 15140122 and 160000-044).

### 4.2. Experimental Animals

Six-week-old male db/db and C57BL6/J mice were provided by GemPharmatech Co., Ltd. (Nanjing, China). The animals were housed in a humidity-controlled room on a 12-h light/dark cycle with free access to standard food and water for three days. Afterwards, db/db mice were fed a high-fat diet to induce diabetes. Fasting blood glucose (FBG) levels above 11.1 mM were considered indicative of successful diabetic status. The diabetic db/db mice were then randomly divided into five groups of six animals each: a model group, a positive control group, and two groups receiving different doses of Nok. Six C57BL6/J mice were used as the normal control group. The normal control and model groups were given equal volumes of saline solution, while the positive control group was treated with metformin at 200 mg/kg/day, and the Nok groups were administered Nok at 25 (Nok-L) or 100 (Nok-H) mg/kg/day via oral gavage. Body weight, food intake, water intake, and FBG were measured weekly.

After four weeks of treatment, all animals were fasted for 12 h, and blood samples were collected from the tail to measure fasting blood glucose (FBG). Blood was then collected from the eyes of all animals into 1.5 mL centrifuge tubes, allowed to stand at room temperature for 2 h, and centrifuged at 3000 rpm for 15 min at 4 °C. The serum was separated, aliquoted, and stored at −80 °C. Serum levels of alanine aminotransferase (ALT), aspartate aminotransferase (AST), low-density lipoprotein (LDL), and triglycerides (TG) were measured using corresponding assay kits. Insulin levels in the serum were determined using an ELISA kit. The homeostasis model assessment of insulin resistance (HOMA-IR) was calculated using the formula: FBG (mM) × insulin (mIU/L)/22.5. The animals were subsequently euthanized, and the liver, kidney, epididymal fat, and pancreas were dissected and weighed. Tissue samples were divided into two parts: one was frozen in liquid nitrogen for further analysis, and the other was fixed in 10% formalin for pathological examination.

All animal experiments were conducted in strict accordance with the “National Institutes of Health Guide for the Care and Use of Laboratory Animals”. The experimental protocol was approved by the Medical Ethics Committee of Hainan Medical University (No. SYXK-2017-0013).

### 4.3. Oral Glucose Tolerance Test (OGTT) and Insulin Tolerance Test (ITT)

OGTT and ITT experiments were conducted during the fourth week of treatment, as our previous methods [21,22].

After 12 h of fasting, the fasting blood glucose (FBG) of all animals was measured as the 0-time point value. A 20% glucose solution was prepared and administered to all animals by oral gavage at a dose of 2 g/kg. Blood glucose levels were measured at 30, 60, 120, and 180 min after glucose administration. The blood glucose data were recorded, and the OGTT curve was plotted.

All animals were fasted for 6 h in the morning, and blood glucose levels were measured as the 0-time point value. Recombinant human insulin was administered via intraperitoneal injection at a dose of 0.5 IU/kg. Blood glucose levels were measured at 30, 60, 90, and 120 min post-insulin injection, and the ITT curve was plotted.

The area under the curve (AUC) for both OGTT and ITT was calculated using GraphPad Prism 8 software.

### 4.4. HE Staining and Histopathological Examination

All mice were euthanized via cervical dislocation, and the liver and pancreas were collected. The tissues were fixed in 10% formalin, embedded in paraffin, and sectioned at 4 μm thickness. The sections were stained with hematoxylin and eosin (HE) and photographed. The extent of hepatic steatosis was scored on a scale of 0 to 5 based on the severity, with the following criteria: 0 = less than 5% of hepatocytes affected; 1 = 5–25% of hepatocytes affected; 2 = 25–50% of hepatocytes affected; 3 = 50–75% of hepatocytes affected; 4 = more than 75% of hepatocytes affected. Pancreatic histopathological changes were scored based on the extent of tissue damage and lesion severity on a scale of 0 to 4: 0 = no lesions; 1 = less than 25% of the pancreatic tissue affected; 2 = 25–50% of the tissue affected; 3 = 50–75% of the tissue affected; 4 = more than 75% of the tissue affected.

### 4.5. Drug Database and Related Target Prediction

The molecular formula and molecular weight data of Nok were obtained from the PubChem database. The target genes of Nok were predicted using the SwissTargetPrediction database and designated as the hypothetical targets of Nok. These hypothetical targets are considered to be drug-related genes.

### 4.6. Prediction of T2DM Related Targets from Online Database

Various gene information related to T2DM was obtained from the GeneCards database, which provides comprehensive data on human genes and genetic disorders. The keywords used to search this database were “Type II diabetes” and “T2DM”, and the results were exported online.

### 4.7. Cell Culture and Treatment

We used AML-12 cells, with DMEM/F12 as the culture medium, supplemented with 10% fetal bovine serum (FBS), 1% ITS Liquid Media Supplement (100×), and 1% penicillin-streptomycin solution. After two stable passages of the AML-12 cells, a cell suspension was prepared and seeded into culture plates and cultured for 24 h. NOK dissolved in dimethyl sulfoxide (DMSO) was then added for co-incubation for 24 h. Subsequent assays were performed.

### 4.8. Cytotoxicity and Glucose Consumption Assay

Cell culture and treatment were carried out as described above, with three replicates for each drug treatment. An equal volume of DMSO (volume ratio of 0.1%) was added to the control group. After 24 h of drug treatment, the supernatant was collected to measure glucose consumption. The culture medium was completely removed, and pre-prepared CCK-8 solution was added to the cells, followed by incubation at 37 °C for 1 h. The OD value at 450 nm was measured using a microplate reader to assess cell viability. Glucose consumption was calculated by subtracting the glucose level in the medium from that of cells treated with the drug or an equal volume of DMSO (volume ratio of 0.1%) for 24 h. After drug treatment, the glucose concentration in the cell supernatant was measured using a glucose assay kit.

### 4.9. Western Blot

Total protein was extracted from AML-12 cells and liver tissue using RIPA lysis buffer containing 1× protease and 1× phosphatase inhibitors. Proteins were separated using SDS-PAGE electrophoresis system and transferred to PVDF membranes. An amount of 5% skim milk was used to block the membranes at 4 °C overnight. After blocking, the membranes were incubated with the primary antibodies (diluted 1:1000 in 5% skim milk/1×TBST) for 2h at room temperature, washed with 1×TBST and then incubated with the secondary antibodies of the same source for 1 h. The membranes were subsequently washed with 1×TBST again and then visualized using the ECL detection kit.

### 4.10. Statistical Analysis

Results were expressed as mean ± S.E.M., with n = 6. Statistical analyses were performed using GraphPad Prism 8 software. Comparisons between two groups were conducted using the Student’s *t*-test, while differences among multiple groups were analyzed using one-way ANOVA. *p* < 0.05 was considered statistically significant.

## 5. Conclusions

In conclusion, Nok may improve insulin resistance, glucose and lipid metabolism disorders, and liver damage in T2DM by targeting both the AMPK and ERK signaling pathways. Network pharmacology helped identify potential molecular targets, which were validated in vivo and in vitro.

## Data Availability

The original contributions presented in the study are included in the article; further inquiries can be directed to the corresponding author(s).
